# Le léiomyome rétro-péritonéal: à propos de 2 cas

**DOI:** 10.11604/pamj.2015.21.7.6603

**Published:** 2015-05-05

**Authors:** Othmane Yddoussalah, Lahyani Mounir, Karmouni Tarik, Elkhader Khalid, Koutani Abdellatif, Ibn Attya Andaloussi Ahmed

**Affiliations:** 1 Centre Universitaire Hospitalier Ibn Sina, Hôpital Ibn Sina, Service d'Urologie B, Rabat, Maroc

**Keywords:** Léiomyome, rétropéritoine, tumeur rétropéritonéale, chirurgie, Leiomyoma, retroperitoneum, retroperitoneal tumor, surgery

## Abstract

Les tumeurs bénignes du muscle lisse sont fréquentes dans le tractus gastro-intestinal et génito-urinaire, et rares au niveau rétro péritonéal. Leur prévalence parmi les tumeurs rétropéritonéales primitives a été estimée entre de 0,5 à 1,2%. Une situation qui conduit à des erreurs de diagnostic. On rapporte dans cet article deux cas de léiomyome rétropéritonéal (LRP) retrouvés chez des femmes âgées entre 47et 54 ans. L'imagerie a mis en évidence une masse rétro-péritonéale, ce qui a motivé une exérèse totale de la tumeur. L'examen anatomopathologique de la pièce opératoire a posé le diagnostic de léiomyome rétro-péritonéal. L’évolution sans récidive était bonne.

## Introduction

Les Léiomyomes sont des tumeurs bénignes qui ont comme origine les cellules musculaires lisses. Ils sont des événements rares qui était reconnus récemment comme des lésions distinctes avec des caractéristiques histologiques similaires à celles du fibrome utérin. Ces tumeurs rétropéritonéales sont en général paucisymptomatiques (douleurs abdominales, lombalgies, perte du poids) ou peuvent encoreêtre totalement asymptomatiques. Poliquin et al [1] ont étudié les caractéristiques d´environ cent cas de fibrome rétropéritonéal. Cette entité rare est généralement difficilement diagnostiquée en préopératoire. Nous rapportons dans cet article deux cas clinique deléiomyome rétro-péritonéal, et analysons, à travers une revue delittérature, les aspects épidémiologiques, diagnostiques et thérapeutiques de cette pathologie.

## Patient et observation

**Figure 1:**
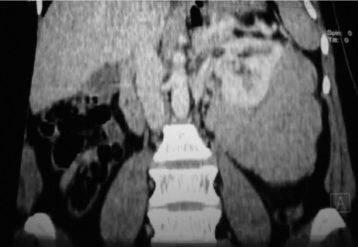
Coupe tomodensitométrique frontale mettant en évidence la masse rétro-péritonéale prenant le rein gauche enmanchon sans l'envahir

**Figure 2:**
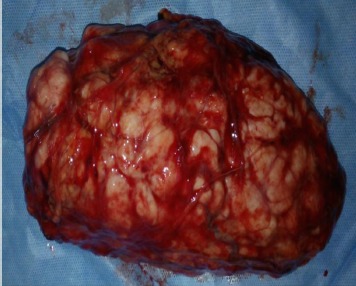
Pièce opératoire de la masse rétro-péritonéale, pesant 800 g, mesurant 19 cm ×9,5 cm × 8,5 cm

**Figure 3:**
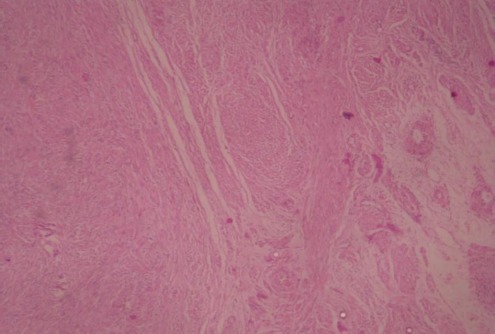
Prolifération à cellules fusiformes (grossissement × 4, hématéine-éosine)

**Figure 4:**
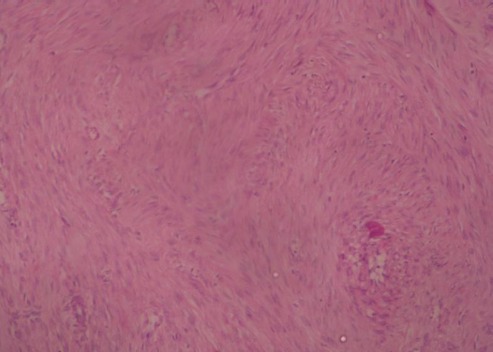
Cellules fusiformes régulières sans activité mitotique (grossissement × 10, hématéine-éosine)

**Cas 1**: Mme E.D, âgée de 43 ans, sans d'antécédent pathologique. Elle avait des cycles encore conservés et réguliers. Elle se plaignait depuis six mois de lombalgies gauches irradiant vers la cuisse gauche, sans troubles urinaire, digestif ou gynécologiqueassociés. A l'examen clinique, la patiente a été apyrétique. Une masse a été palpée au niveau du flanc gauche s’étendant sur 6 cm au-dessous du rebord costal, de consistance solide, et fixe au plan profond. La tomodensitométrie a objectivé une masse rétro-péritonéale, de 18 cm de grand axe, dont la densité tissulaire a été, estimée à 50 unités Hounsfield (UH) sans injection, se rehaussant après injection du produit de contraste. Cette masse a été en contact intime avec le rein gauche qui refoulé vers l'avant. Elle a été également en rapport intime avec la face postéro-inférieure de la rate (Figure 1). Il n'y a pas eu d'envahissement rénal ni splénique. Aucune adénopathie n'a été visible. Sur le plan biologique, la patiente a eu un taux d'hémoglobine à 13 g/dl, des leucocytes à 5400/mm3, une fonction rénale normale avec une créatininémie à 6,7 mg/L. L'abord chirurgical était par voie sous-costale gauche. Après décollement de l'angle colique gauche, une masse rétro-péritonéale a été découverte, d'aspect blanchâtre, mobile, adhérente à la face postérieure du rein. Cette masse a été libérée du rein. Après exérèse totale de la masse (Figure 2), une brèche capsulaire rénale, occasionnée lors de la libération de la masse, a été fermée. Les suites opératoires ont été simples. A l'examen macroscopique, la masse est ferme, d'aspect homogène, nodulaire, de couleur blanchâtre, sans remaniements nécrotico-hémorragique ou kystique. L'examen microscopique a mis en évidence une prolifération tumorale fuso-cellulaire d'architecture nodulaire, faite de faisceaux entrecroisés (Figure 3). Les cellules tumorales ont un noyau allongé, vésiculeux, avec un fin nucléole dépourvu d'atypies, et un cytoplasme éosinophile mal limité. Les mitoses ont été estimées à une mitose par dix champs au fort grossissement. Il n'y a pas eu de nécrose (Figure 4). L’étude immuno-histochimique a objectivé un marquage positif diffus des cellules tumorales aux anticorps anti-caldesmone et anti-AML, et positif à l'anticorps anti-P100. Le marquage des cellules tumorales à l'anticorps anti-Ki67 aété estimé à 5%. Or, le marquage s'est révélé négatif à l'anticorps anti-CD117. Cette analyse morphologique et immunohistochimiques a conclu à un léiomyome de type génital. La patiente a été ensuite adressée pour une évaluation gynécologique, qui n'a pas décelé de léiomyome utérin, et le frottis cervico-vaginal a été normal. La patiente a été revue en consultation à un mois, six mois et un an; son examen clinique a été normal et la tomodensitométrie abdomino-pelvienne réalisée après un an n'a pas montré de récidive.

**Figure 5:**
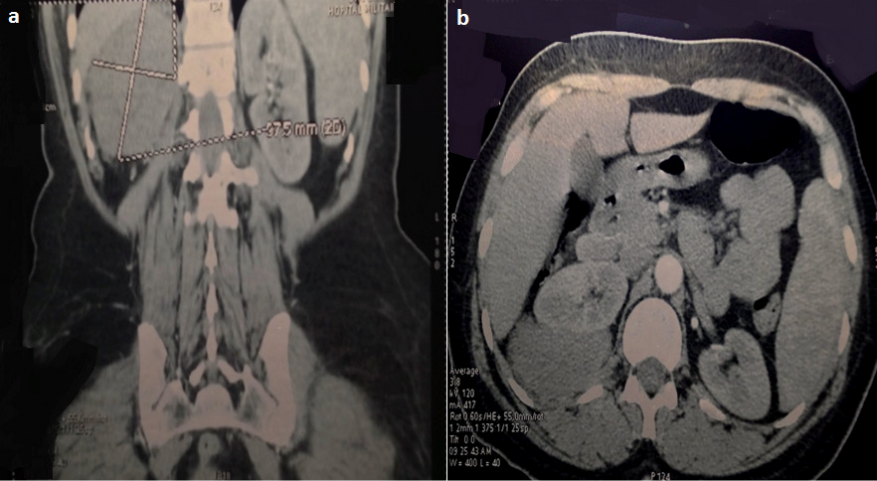
TDM abdominale montrant un processus tissulaire ovalaire de 97 × 47 mm de grand axe rétropéritonéale et hypodense (a,b)

**Cas 2**: Mme B.M âgée de 54 ans, sans antécédents particuliers, a présenté des lombalgies droites, sans hématurie ou autres manifestations urologiques, le tout évoluant dans un contexte d apyrexie. L examen clinique était strictement normal. L’échographie abdominale a montré la présence d'une masse tissulaire hétérogène inter hépato rénal avec un utérus myomateux. La TDM abdominale a objectivé une masse rétropéritonéale hypodense ovalaire de 9 cm de grand axe et de 44 UH densité. Cette lésion était en rapport étroit avec le bord postérieur du pôle supérieur du rein droit (Figure 5). L'exploration chirurgicale par lombotomie droite avec résection de la onzième côte, découvre une masse solide retrorénale droite. On réalise alors une exérèse complète emportant un petit moignon adhérentiel du rein droit. Les suites opératoires ont été simples. Macroscopiquement, la volumineuse masse était de couleur jaunâtre, fasciculée avec présence de bandes fibreuses blanchâtres et dures. A L'examen histologique les coupes analysées montrent une large prolifération tumorale bégnine, des fascicules faits de cellules musculaires lisses à noyau fusiforme et régulier. Cette prolifération est dissociée par un stroma fibreux. Le tout est en faveur d'un léiomyome sans signes de malignité.

## Discussion

Les fibromes utérins sont les tumeurs pelviennes solides les plus communes chez les femmes [2] et sont présentes dans environ 80% de toutes les hystérectomies [3]. Le Léiomyome touche fréquemment les patientes dans la quatrièmeet cinquième décades de la vie. Le corps de l´utérus est la localisation la plus fréquente. Leur étiologie est encore mal connue, mais il est admis que les estrogènes et la progestérone sont impliqués dans laprolifération tumorale, tant que les fibromes apparaissent rarement avant la ménarche, et régressent après la ménopause [4]. Les présentations des léiomyomes extra-utérins les plusrapportées dans la littérature sont: les léiomyomes métastatiques bénins, la léiomyomatose péritonéale disséminée, la léiomyomatose intraveineuse, la léiomyomatose parasitaire etles masses rétropéritonéales [5]. Les LRPqui ont été décritsdans la littérature présentent une prévalence parmi les tumeurs rétropéritonéales primitives estimée entre 0,5 à 1,2% [6, 7]. Ces tumeurs sont apparues principalement chez des femmes, dans le quatrième-cinquième décennies de vie. Les aspects histologiques sontsimilaires à celle des léiomyomes utérins [8]. En ce qui concerne leur étiopathogénie, il est difficile de savoir si les LRP sont des lésions primaires ou métastatiques synchrones, mais encore s'ils proviennent des éléments musculaires lisses sensibles aux hormones [7]. Stutterecker et al. Pense que les vestiges embryonnaires des canaux de Muller ou Wolff pourraient être à l'origine du développement des LRP [9]. Kang et al. [10] ont suggéré l'origine multifocale primairedes LRP. Kho et Nezhat [11] ont proposé une origine « iatrogène » des LRP en analysant une série de cas de léiomyomes extra-utérins. Ils ont constaté que 83% de leur série de cas avaient eu une chirurgie abdominale, 67% avaient eu des myomectomies utérines, dont la plupart par laparoscopie avec morcellation. Le diagnostic préopératoire n'est pas toujours évident à cause de la rareté de cette tumeur, d'une part, et de la présentation clinique non spécifique, d'autre part. Les symptômes les plus fréquents comprennent des douleurs abdominales, fatigue, lombalgies, dyspareunie et des signes en rapport avec la compression des organes adjacents. Plus de 40% des cas de LRP ont eu un léiomyome utérin concomitant ou un antécédent récent d'hystérectomie pour fibrome utérin [1, 12]. La prise en charge chirurgicale est basée sur l'exérèse complète de la masse tumorale; réalisée dans la majorité des cas par chirurgie ouverte. Kondo et al. [13] ont rapporté un cas de fibrome rétropéritonéale opérée par coelioscopie. L'hystérectomie peut être réalisée dans certains cas, en fonction de l’âge, la symptomatologie et l'importance du myome utérin associé.

## Conclusion

Les LRP sont des tumeurs rares qu'on doit toujours évoquer lors de toute évaluation d'une masse rétropéritonéale. L'imagerie est cruciale pour préciser l'extension locale mais seule l'analyse histologique et immunohistochimique de la pièce d'exérèse permettra d'assoir le diagnostic positif. La surveillance clinico-radiologique s'impose malgré le bon pronostic.
